# 9-Bromo-9-borafluorene

**DOI:** 10.1107/S1600536810001625

**Published:** 2010-01-23

**Authors:** Alexander Hübner, Hans-Wolfram Lerner, Matthias Wagner, Michael Bolte

**Affiliations:** a Institut für Anorganische Chemie der Goethe-Universität Frankfurt, Max-von-Laue-Strasse 7, D-60438 Frankfurt am Main, Germany

## Abstract

The title compound, C_12_H_8_BBr, crystallizes with three essentially planar mol­ecules (r.m.s. deviations = 0.018, 0.020 and 0.021Å) in the asymmetric unit: since the title compound is rigid, there are no conformational differences between these three mol­ecules. The crystal packing resembles a herringbone pattern.

## Related literature

For the synthesis of 9-ferrocenyl-9-borafluorene derivatives, see: Kaufmann *et al.* (2008 ▶). The title compound was obtained by treatment of 9,9-dimethyl-9-silafluorene (Mewes *et al.*, 2009 ▶) with BBr_3_ following a modified literature procedure (Gross *et al.*, 1987 ▶).
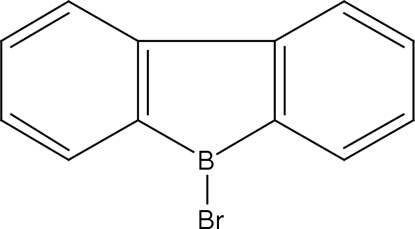


## Experimental

### 

#### Crystal data

C_12_H_8_BBr
*M* *_r_* = 242.90
Orthorhombic, 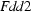

*a* = 34.939 (3) Å
*b* = 85.482 (4) Å
*c* = 3.9672 (2) Å
*V* = 11848.7 (13) Å^3^
*Z* = 48
Mo *K*α radiation
μ = 4.11 mm^−1^
*T* = 173 K
0.19 × 0.03 × 0.03 mm

#### Data collection

Stoe IPDS II two-circle diffractometer
Absorption correction: multi-scan (*MULABS*; Spek, 2009 ▶; Blessing, 1995 ▶) *T*_min_ = 0.509, *T*_max_ = 0.887
20875 measured reflections
5204 independent reflections
3565 reflections with *I* > 2σ(*I*)
*R*_int_ = 0.080

#### Refinement

*R*[*F* ^2^ > 2σ(*F* ^2^)] = 0.051
*wR*(*F* ^2^) = 0.100
*S* = 0.86
5204 reflections
380 parameters
1 restraint
H-atom parameters constrained
Δρ_max_ = 0.46 e Å^−3^
Δρ_min_ = −0.61 e Å^−3^
Absolute structure: Flack (1983 ▶), 2183 Friedel pairs
Flack parameter: 0.320 (19)

### 

Data collection: *X-AREA* (Stoe & Cie, 2001 ▶); cell refinement: *X-AREA*; data reduction: *X-AREA*; program(s) used to solve structure: *SHELXS97* (Sheldrick, 2008 ▶); program(s) used to refine structure: *SHELXL97* (Sheldrick, 2008 ▶); molecular graphics: *XP* (Sheldrick, 2008 ▶); software used to prepare material for publication: *SHELXL97* and *PLATON* (Spek, 2009 ▶).

## Supplementary Material

Crystal structure: contains datablocks I, global. DOI: 10.1107/S1600536810001625/fk2010sup1.cif

Structure factors: contains datablocks I. DOI: 10.1107/S1600536810001625/fk2010Isup2.hkl

Additional supplementary materials:  crystallographic information; 3D view; checkCIF report

## Figures and Tables

**Fig. 1.**
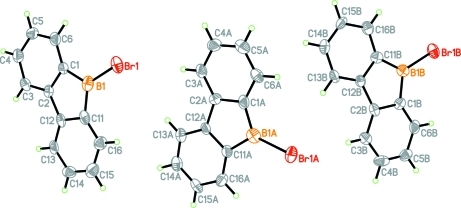
A view of the three molecules in the asymmetric unit of the title compound, with the atom-numbering scheme. Displacement ellipsoids are drawn at the 50% probability level and H atoms are shown as small spheres of arbitrary radii.

**Fig. 2.**
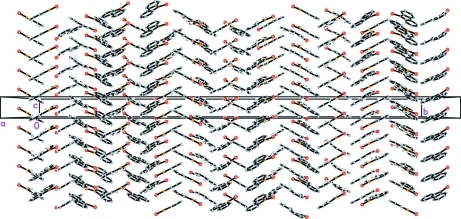
Packing diagram with view onto the bc plane. Hydrogen atoms omitted for clarity.

**Fig. 3.**
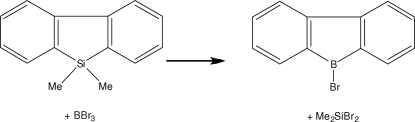
The formation of the title compound.

## supplementary crystallographic information

### Comment

Polyferrocenylenes with bridging elements E*R_x_* (e. g. E*R_x_* = B*R*, Si*R*_2_, Sn*R*_2_, P*R*, S) represent an important class of processable metal-containing polymers with applications ranging from molecular electronics to the preparation of magnetic ceramics. We are currently interested in negatively charged polyferrocenylenes with borate linkers. To this end, we have synthesized 9-ferrocenyl-9-borafluorene derivatives (Kaufmann *et al.*, 2008) as building blocks for such polymers. Herein, we describe the preparation and solid state structure of 9-bromo-9-borafluorene (C_12_H_8_BBr), which we have already used as a starting material in the synthesis of 9-ferrocenyl-9-borafluorene derivatives. The title compound was obtained by treatment of 9,9-dimethyl-9-silafluorene (Mewes *et al.*, 2009) with BBr_3_ following a modified literature procedure (Gross *et al.*, 1987), as indicated in the equation (Fig. 3).

The title compound (Fig. 1) crystallizes with three essentially planar molecules (r.m.s. deviation = 0.018 Å, 0.020 Å, 0.021 Å) in the asymmetric unit. Since the title compound features a rigid molecule, there are no conformational differences between these three molecules.

The crystal packing resembles a herring bone pattern (Fig. 2).

### Experimental

A mixture of 9,9-dimethyl-9-silafluorene (0.52 g, 2.47 mmol) and BBr_3_ (0.6 ml, 1.59 g, 6.35 mmol) was heated in a sealed ampoule for 52 h at 328 K. After removal of all volatiles in vacuo, X-ray quality crystals of the title compound were obtained from a hexane solution at room temperature (yield: 0.60 g, 2.47 mmol, 100 °).

### Refinement

Hydrogen atoms were located in a difference Fourier map but they were included in calculated positions [C—H = 0.95 Å] and refined as riding [*U*_iso_(H) = 1.2*U*_eq_(C)]. The crystal turned out to be a racemic twin with a ratio of of 0.680 (19)/0.320 (19) for the two twin components.

### Crystal data

**Table:**

C_12_H_8_BBr	*F*(000) = 5760
*M**_r_* = 242.90	*D*_x_ = 1.634 Mg m^−^^3^
Orthorhombic, *F**d**d*2	Mo *K*α radiation, λ = 0.71073 Å
Hall symbol: F 2 -2d	Cell parameters from 9350 reflections
*a* = 34.939 (3) Å	θ = 2.4–25.3°
*b* = 85.482 (4) Å	µ = 4.11 mm^−^^1^
*c* = 3.9672 (2) Å	*T* = 173 K
*V* = 11848.7 (13) Å^3^	Needle, yellow
*Z* = 48	0.19 × 0.03 × 0.03 mm

### Data collection

**Table:**

Stoe IPDS II two-circle diffractometer	5204 independent reflections
Radiation source: fine-focus sealed tube	3565 reflections with *I* > 2σ(*I*)
graphite	*R*_int_ = 0.080
ω scans	θ_max_ = 25.1°, θ_min_ = 2.2°
Absorption correction: multi-scan (*MULABS*; Spek, 2009; Blessing, 1995)	*h* = −40→40
*T*_min_ = 0.509, *T*_max_ = 0.887	*k* = −100→90
20875 measured reflections	*l* = −4→4

### Refinement

**Table:**

Refinement on *F*^2^	Secondary atom site location: difference Fourier map
Least-squares matrix: full	Hydrogen site location: inferred from neighbouring sites
*R*[*F*^2^ > 2σ(*F*^2^)] = 0.051	H-atom parameters constrained
*wR*(*F*^2^) = 0.100	*w* = 1/[σ^2^(*F*_o_^2^) + (0.0328*P*)^2^] where *P* = (*F*_o_^2^ + 2*F*_c_^2^)/3
*S* = 0.86	(Δ/σ)_max_ = 0.002
5204 reflections	Δρ_max_ = 0.46 e Å^−^^3^
380 parameters	Δρ_min_ = −0.61 e Å^−^^3^
1 restraint	Absolute structure: Flack (1983), 2183 Friedel pairs
Primary atom site location: structure-invariant direct methods	Flack parameter: 0.320 (19)

### Special details

**Table:**

Geometry. All e.s.d.'s (except the e.s.d. in the dihedral angle between two l.s. planes) are estimated using the full covariance matrix. The cell e.s.d.'s are taken into account individually in the estimation of e.s.d.'s in distances, angles and torsion angles; correlations between e.s.d.'s in cell parameters are only used when they are defined by crystal symmetry. An approximate (isotropic) treatment of cell e.s.d.'s is used for estimating e.s.d.'s involving l.s. planes.
Refinement. Refinement of *F*^2^ against ALL reflections. The weighted *R*-factor *wR* and goodness of fit *S* are based on *F*^2^, conventional *R*-factors *R* are based on *F*, with *F* set to zero for negative *F*^2^. The threshold expression of *F*^2^ > σ(*F*^2^) is used only for calculating *R*-factors(gt) *etc*. and is not relevant to the choice of reflections for refinement. *R*-factors based on *F*^2^ are statistically about twice as large as those based on *F*, and *R*- factors based on ALL data will be even larger.

### Fractional atomic coordinates and isotropic or equivalent isotropic displacement parameters (Å^2^)

**Table:**

	*x*	*y*	*z*	*U*_iso_*/*U*_eq_	
Br1	0.57091 (3)	0.512885 (9)	0.9579 (2)	0.0389 (2)	
B1	0.5914 (3)	0.49518 (11)	0.727 (2)	0.032 (2)	
C1	0.5689 (2)	0.48119 (9)	0.5713 (18)	0.0265 (18)	
C2	0.5971 (2)	0.47105 (9)	0.434 (2)	0.0265 (17)	
C3	0.5857 (2)	0.45687 (10)	0.277 (2)	0.033 (2)	
H3	0.6042	0.4499	0.1877	0.039*	
C4	0.5470 (2)	0.45348 (10)	0.258 (2)	0.036 (2)	
H4	0.5389	0.4440	0.1535	0.043*	
C5	0.5196 (2)	0.46368 (10)	0.390 (2)	0.039 (2)	
H5	0.4933	0.4611	0.3714	0.047*	
C6	0.5304 (2)	0.47751 (9)	0.546 (2)	0.034 (2)	
H6	0.5115	0.4844	0.6357	0.041*	
C11	0.6343 (2)	0.49174 (9)	0.656 (2)	0.0298 (18)	
C12	0.6363 (2)	0.47732 (8)	0.486 (2)	0.0286 (18)	
C13	0.6714 (2)	0.47142 (10)	0.378 (2)	0.035 (2)	
H13	0.6727	0.4618	0.2579	0.042*	
C14	0.7045 (2)	0.47980 (10)	0.449 (2)	0.036 (2)	
H14	0.7286	0.4758	0.3772	0.044*	
C15	0.7029 (3)	0.49375 (11)	0.621 (2)	0.043 (2)	
H15	0.7260	0.4993	0.6677	0.052*	
C16	0.6681 (2)	0.49993 (10)	0.729 (2)	0.036 (2)	
H16	0.6672	0.5095	0.8495	0.044*	
Br1A	0.69438 (3)	0.598421 (10)	1.03380 (19)	0.0392 (2)	
B1A	0.6694 (3)	0.58003 (12)	0.884 (2)	0.031 (2)	
C1A	0.6268 (2)	0.57608 (9)	0.914 (2)	0.0327 (19)	
C2A	0.6208 (2)	0.56131 (9)	0.758 (2)	0.0249 (17)	
C3A	0.5844 (2)	0.55494 (9)	0.739 (2)	0.033 (2)	
H3A	0.5804	0.5452	0.6286	0.040*	
C4A	0.5537 (2)	0.56287 (10)	0.882 (2)	0.038 (2)	
H4A	0.5287	0.5585	0.8700	0.046*	
C5A	0.5592 (2)	0.57729 (10)	1.045 (2)	0.038 (2)	
H5A	0.5382	0.5825	1.1473	0.046*	
C6A	0.5955 (2)	0.58378 (10)	1.055 (2)	0.034 (2)	
H6A	0.5991	0.5937	1.1591	0.040*	
C11A	0.6880 (2)	0.56560 (8)	0.705 (2)	0.0293 (18)	
C12A	0.6578 (2)	0.55499 (9)	0.631 (2)	0.0257 (19)	
C13A	0.6650 (2)	0.54106 (9)	0.464 (2)	0.0334 (19)	
H13A	0.6447	0.5341	0.4107	0.040*	
C14A	0.7021 (3)	0.53746 (10)	0.375 (2)	0.035 (2)	
H14A	0.7073	0.5277	0.2690	0.041*	
C15A	0.7320 (2)	0.54767 (9)	0.438 (3)	0.038 (2)	
H15A	0.7572	0.5451	0.3690	0.045*	
C16A	0.7247 (2)	0.56172 (10)	0.603 (2)	0.036 (2)	
H16A	0.7452	0.5687	0.6472	0.043*	
Br1B	0.57408 (3)	0.680334 (9)	−0.0716 (3)	0.0384 (2)	
B1B	0.5983 (3)	0.66204 (11)	0.112 (2)	0.030 (2)	
C1B	0.6416 (2)	0.65923 (9)	0.1237 (19)	0.027 (2)	
C2B	0.6472 (2)	0.64463 (9)	0.291 (2)	0.0259 (18)	
C3B	0.6835 (2)	0.63877 (11)	0.351 (2)	0.033 (2)	
H3B	0.6869	0.6292	0.4701	0.040*	
C4B	0.7149 (2)	0.64705 (11)	0.235 (2)	0.038 (2)	
H4B	0.7399	0.6430	0.2698	0.046*	
C5B	0.7102 (2)	0.66116 (10)	0.068 (2)	0.034 (2)	
H5B	0.7320	0.6667	−0.0107	0.041*	
C6B	0.6739 (2)	0.66719 (9)	0.014 (2)	0.035 (2)	
H6B	0.6711	0.6769	−0.0992	0.042*	
C11B	0.5793 (2)	0.64763 (9)	0.276 (2)	0.0284 (18)	
C12B	0.6090 (2)	0.63775 (9)	0.3814 (19)	0.0258 (19)	
C13B	0.6010 (2)	0.62372 (9)	0.542 (2)	0.0292 (18)	
H13B	0.6212	0.6171	0.6162	0.035*	
C14B	0.5630 (2)	0.61940 (10)	0.593 (2)	0.032 (2)	
H14B	0.5572	0.6097	0.6970	0.039*	
C15B	0.5334 (2)	0.62921 (9)	0.493 (2)	0.0304 (19)	
H15B	0.5076	0.6262	0.5320	0.036*	
C16B	0.5409 (2)	0.64345 (10)	0.336 (2)	0.033 (2)	
H16B	0.5206	0.6502	0.2718	0.040*	

### Atomic displacement parameters (Å^2^)

**Table:**

	*U*^11^	*U*^22^	*U*^33^	*U*^12^	*U*^13^	*U*^23^
Br1	0.0557 (5)	0.0268 (4)	0.0342 (5)	0.0070 (4)	0.0007 (5)	−0.0021 (5)
B1	0.048 (6)	0.031 (5)	0.017 (5)	−0.004 (4)	−0.004 (4)	0.007 (4)
C1	0.030 (4)	0.022 (4)	0.028 (4)	0.000 (3)	−0.001 (4)	0.002 (3)
C2	0.034 (4)	0.021 (4)	0.025 (4)	−0.003 (3)	−0.002 (4)	0.002 (4)
C3	0.039 (5)	0.029 (4)	0.031 (5)	0.007 (4)	−0.004 (4)	0.004 (4)
C4	0.043 (5)	0.026 (4)	0.039 (6)	−0.001 (4)	−0.008 (5)	0.001 (4)
C5	0.029 (4)	0.038 (5)	0.049 (6)	−0.003 (4)	−0.006 (4)	0.010 (5)
C6	0.041 (5)	0.028 (4)	0.033 (5)	0.002 (4)	0.006 (4)	0.007 (4)
C11	0.042 (5)	0.027 (4)	0.020 (4)	0.004 (3)	0.000 (4)	0.010 (4)
C12	0.042 (5)	0.020 (4)	0.023 (5)	0.004 (3)	−0.002 (4)	0.006 (4)
C13	0.041 (5)	0.024 (4)	0.039 (6)	0.003 (4)	0.005 (4)	0.016 (4)
C14	0.029 (5)	0.046 (5)	0.034 (5)	0.003 (4)	0.004 (4)	0.009 (5)
C15	0.045 (6)	0.045 (6)	0.039 (6)	−0.012 (4)	−0.012 (4)	0.017 (5)
C16	0.038 (5)	0.035 (4)	0.037 (5)	−0.004 (3)	−0.005 (5)	−0.001 (5)
Br1A	0.0505 (5)	0.0286 (4)	0.0386 (5)	−0.0069 (4)	−0.0025 (4)	−0.0062 (4)
B1A	0.043 (6)	0.039 (5)	0.012 (5)	0.004 (4)	−0.004 (4)	0.004 (4)
C1A	0.040 (5)	0.023 (4)	0.036 (5)	0.001 (3)	−0.003 (4)	0.010 (4)
C2A	0.026 (4)	0.032 (4)	0.017 (4)	−0.002 (3)	−0.003 (3)	0.007 (4)
C3A	0.039 (5)	0.025 (4)	0.034 (5)	0.003 (3)	−0.015 (4)	0.006 (4)
C4A	0.031 (4)	0.041 (5)	0.042 (6)	−0.002 (4)	0.007 (4)	0.008 (4)
C5A	0.040 (5)	0.043 (5)	0.032 (5)	0.012 (4)	0.007 (4)	0.009 (4)
C6A	0.039 (5)	0.032 (4)	0.029 (5)	0.010 (4)	−0.003 (4)	0.004 (4)
C11A	0.032 (4)	0.022 (4)	0.034 (4)	0.001 (3)	−0.003 (4)	−0.002 (4)
C12A	0.029 (4)	0.020 (4)	0.028 (5)	0.003 (3)	−0.005 (4)	0.003 (3)
C13A	0.044 (5)	0.023 (4)	0.033 (5)	0.001 (4)	−0.002 (4)	−0.002 (4)
C14A	0.050 (5)	0.027 (4)	0.027 (5)	0.002 (4)	0.003 (4)	−0.003 (4)
C15A	0.044 (5)	0.028 (4)	0.040 (5)	0.015 (4)	0.002 (5)	−0.001 (5)
C16A	0.035 (5)	0.031 (5)	0.043 (6)	−0.008 (4)	−0.001 (4)	0.002 (4)
Br1B	0.0501 (5)	0.0283 (4)	0.0369 (5)	0.0083 (4)	0.0004 (4)	0.0072 (5)
B1B	0.031 (5)	0.034 (5)	0.026 (6)	0.002 (4)	−0.003 (4)	0.000 (4)
C1B	0.031 (4)	0.029 (4)	0.023 (5)	−0.004 (3)	−0.004 (3)	0.003 (3)
C2B	0.036 (4)	0.023 (4)	0.018 (4)	0.005 (3)	−0.002 (4)	0.000 (4)
C3B	0.033 (5)	0.033 (5)	0.033 (5)	0.004 (4)	0.000 (4)	−0.001 (4)
C4B	0.024 (4)	0.059 (6)	0.032 (5)	0.003 (4)	−0.003 (4)	−0.013 (5)
C5B	0.032 (5)	0.040 (5)	0.030 (5)	−0.007 (4)	0.000 (4)	−0.008 (4)
C6B	0.043 (5)	0.027 (4)	0.035 (5)	−0.004 (4)	0.005 (5)	−0.011 (4)
C11B	0.029 (4)	0.034 (4)	0.022 (4)	0.001 (3)	−0.003 (4)	0.000 (4)
C12B	0.031 (4)	0.023 (4)	0.024 (5)	0.001 (3)	0.000 (4)	−0.005 (4)
C13B	0.035 (5)	0.020 (4)	0.033 (5)	0.006 (3)	−0.005 (4)	0.000 (4)
C14B	0.046 (5)	0.023 (4)	0.028 (5)	−0.002 (4)	0.004 (4)	0.001 (3)
C15B	0.030 (4)	0.027 (4)	0.034 (5)	−0.012 (4)	0.002 (4)	−0.005 (4)
C16B	0.040 (5)	0.030 (4)	0.029 (5)	0.004 (4)	−0.002 (4)	−0.002 (4)

### Geometric parameters (Å, °)

**Table:**

Br1—B1	1.909 (10)	C5A—H5A	0.9500
B1—C11	1.554 (12)	C6A—H6A	0.9500
B1—C1	1.557 (12)	C11A—C16A	1.384 (11)
C1—C6	1.386 (10)	C11A—C12A	1.421 (10)
C1—C2	1.422 (11)	C12A—C13A	1.387 (11)
C2—C3	1.420 (11)	C13A—C14A	1.377 (12)
C2—C12	1.482 (10)	C13A—H13A	0.9500
C3—C4	1.387 (11)	C14A—C15A	1.382 (12)
C3—H3	0.9500	C14A—H14A	0.9500
C4—C5	1.394 (12)	C15A—C16A	1.392 (12)
C4—H4	0.9500	C15A—H15A	0.9500
C5—C6	1.387 (12)	C16A—H16A	0.9500
C5—H5	0.9500	Br1B—B1B	1.920 (10)
C6—H6	0.9500	B1B—C1B	1.534 (12)
C11—C16	1.402 (11)	B1B—C11B	1.543 (12)
C11—C12	1.408 (11)	C1B—C6B	1.387 (11)
C12—C13	1.396 (11)	C1B—C2B	1.428 (11)
C13—C14	1.388 (12)	C2B—C3B	1.384 (11)
C13—H13	0.9500	C2B—C12B	1.504 (11)
C14—C15	1.376 (13)	C3B—C4B	1.383 (12)
C14—H14	0.9500	C3B—H3B	0.9500
C15—C16	1.395 (13)	C4B—C5B	1.386 (12)
C15—H15	0.9500	C4B—H4B	0.9500
C16—H16	0.9500	C5B—C6B	1.385 (12)
Br1A—B1A	1.892 (10)	C5B—H5B	0.9500
B1A—C1A	1.533 (13)	C6B—H6B	0.9500
B1A—C11A	1.565 (12)	C11B—C12B	1.402 (10)
C1A—C6A	1.392 (11)	C11B—C16B	1.408 (11)
C1A—C2A	1.421 (11)	C12B—C13B	1.386 (11)
C2A—C3A	1.387 (10)	C13B—C14B	1.396 (11)
C2A—C12A	1.488 (11)	C13B—H13B	0.9500
C3A—C4A	1.390 (12)	C14B—C15B	1.389 (11)
C3A—H3A	0.9500	C14B—H14B	0.9500
C4A—C5A	1.404 (13)	C15B—C16B	1.391 (11)
C4A—H4A	0.9500	C15B—H15B	0.9500
C5A—C6A	1.386 (12)	C16B—H16B	0.9500
			
C11—B1—C1	105.7 (7)	C1A—C6A—H6A	119.4
C11—B1—Br1	126.7 (6)	C16A—C11A—C12A	118.4 (7)
C1—B1—Br1	127.6 (6)	C16A—C11A—B1A	134.8 (7)
C6—C1—C2	120.5 (7)	C12A—C11A—B1A	106.8 (7)
C6—C1—B1	133.8 (7)	C13A—C12A—C11A	120.7 (7)
C2—C1—B1	105.7 (7)	C13A—C12A—C2A	129.2 (7)
C3—C2—C1	119.5 (7)	C11A—C12A—C2A	110.1 (7)
C3—C2—C12	129.0 (7)	C14A—C13A—C12A	119.0 (8)
C1—C2—C12	111.5 (7)	C14A—C13A—H13A	120.5
C4—C3—C2	118.5 (7)	C12A—C13A—H13A	120.5
C4—C3—H3	120.7	C13A—C14A—C15A	121.6 (8)
C2—C3—H3	120.7	C13A—C14A—H14A	119.2
C3—C4—C5	121.1 (8)	C15A—C14A—H14A	119.2
C3—C4—H4	119.4	C14A—C15A—C16A	119.4 (8)
C5—C4—H4	119.4	C14A—C15A—H15A	120.3
C6—C5—C4	121.1 (8)	C16A—C15A—H15A	120.3
C6—C5—H5	119.5	C11A—C16A—C15A	120.8 (8)
C4—C5—H5	119.5	C11A—C16A—H16A	119.6
C1—C6—C5	119.2 (8)	C15A—C16A—H16A	119.6
C1—C6—H6	120.4	C1B—B1B—C11B	106.6 (7)
C5—C6—H6	120.4	C1B—B1B—Br1B	125.0 (6)
C16—C11—C12	119.7 (7)	C11B—B1B—Br1B	128.3 (6)
C16—C11—B1	132.9 (8)	C6B—C1B—C2B	117.5 (8)
C12—C11—B1	107.4 (7)	C6B—C1B—B1B	135.7 (8)
C13—C12—C11	120.4 (7)	C2B—C1B—B1B	106.7 (7)
C13—C12—C2	129.7 (7)	C3B—C2B—C1B	121.5 (8)
C11—C12—C2	109.8 (7)	C3B—C2B—C12B	129.2 (7)
C14—C13—C12	119.0 (8)	C1B—C2B—C12B	109.2 (7)
C14—C13—H13	120.5	C4B—C3B—C2B	118.9 (8)
C12—C13—H13	120.5	C4B—C3B—H3B	120.6
C15—C14—C13	121.0 (8)	C2B—C3B—H3B	120.6
C15—C14—H14	119.5	C3B—C4B—C5B	120.7 (8)
C13—C14—H14	119.5	C3B—C4B—H4B	119.6
C14—C15—C16	121.0 (8)	C5B—C4B—H4B	119.6
C14—C15—H15	119.5	C4B—C5B—C6B	120.4 (8)
C16—C15—H15	119.5	C4B—C5B—H5B	119.8
C15—C16—C11	118.9 (8)	C6B—C5B—H5B	119.8
C15—C16—H16	120.5	C5B—C6B—C1B	120.9 (8)
C11—C16—H16	120.5	C5B—C6B—H6B	119.5
C1A—B1A—C11A	105.3 (7)	C1B—C6B—H6B	119.5
C1A—B1A—Br1A	127.3 (6)	C12B—C11B—C16B	120.0 (7)
C11A—B1A—Br1A	127.4 (6)	C12B—C11B—B1B	106.8 (6)
C6A—C1A—C2A	118.6 (7)	C16B—C11B—B1B	133.2 (7)
C6A—C1A—B1A	133.7 (8)	C13B—C12B—C11B	120.7 (7)
C2A—C1A—B1A	107.7 (7)	C13B—C12B—C2B	128.7 (7)
C3A—C2A—C1A	120.5 (7)	C11B—C12B—C2B	110.6 (7)
C3A—C2A—C12A	129.4 (7)	C12B—C13B—C14B	119.1 (7)
C1A—C2A—C12A	110.1 (6)	C12B—C13B—H13B	120.4
C2A—C3A—C4A	119.6 (8)	C14B—C13B—H13B	120.4
C2A—C3A—H3A	120.2	C15B—C14B—C13B	120.5 (7)
C4A—C3A—H3A	120.2	C15B—C14B—H14B	119.8
C3A—C4A—C5A	120.8 (8)	C13B—C14B—H14B	119.8
C3A—C4A—H4A	119.6	C14B—C15B—C16B	121.1 (7)
C5A—C4A—H4A	119.6	C14B—C15B—H15B	119.5
C6A—C5A—C4A	119.2 (8)	C16B—C15B—H15B	119.5
C6A—C5A—H5A	120.4	C15B—C16B—C11B	118.6 (7)
C4A—C5A—H5A	120.4	C15B—C16B—H16B	120.7
C5A—C6A—C1A	121.3 (8)	C11B—C16B—H16B	120.7
C5A—C6A—H6A	119.4		
			
C11—B1—C1—C6	−178.9 (8)	C1A—B1A—C11A—C12A	2.1 (9)
Br1—B1—C1—C6	1.4 (14)	Br1A—B1A—C11A—C12A	−178.0 (6)
C11—B1—C1—C2	0.7 (8)	C16A—C11A—C12A—C13A	0.4 (12)
Br1—B1—C1—C2	−179.0 (6)	B1A—C11A—C12A—C13A	178.6 (8)
C6—C1—C2—C3	−1.4 (12)	C16A—C11A—C12A—C2A	−179.7 (8)
B1—C1—C2—C3	178.9 (8)	B1A—C11A—C12A—C2A	−1.4 (9)
C6—C1—C2—C12	179.4 (7)	C3A—C2A—C12A—C13A	−0.6 (14)
B1—C1—C2—C12	−0.3 (9)	C1A—C2A—C12A—C13A	−179.9 (8)
C1—C2—C3—C4	1.1 (12)	C3A—C2A—C12A—C11A	179.5 (8)
C12—C2—C3—C4	−179.9 (8)	C1A—C2A—C12A—C11A	0.2 (9)
C2—C3—C4—C5	−0.1 (13)	C11A—C12A—C13A—C14A	1.4 (13)
C3—C4—C5—C6	−0.5 (14)	C2A—C12A—C13A—C14A	−178.6 (8)
C2—C1—C6—C5	0.8 (12)	C12A—C13A—C14A—C15A	−2.5 (13)
B1—C1—C6—C5	−179.6 (8)	C13A—C14A—C15A—C16A	2.0 (13)
C4—C5—C6—C1	0.2 (13)	C12A—C11A—C16A—C15A	−1.0 (13)
C1—B1—C11—C16	179.1 (9)	B1A—C11A—C16A—C15A	−178.6 (9)
Br1—B1—C11—C16	−1.2 (14)	C14A—C15A—C16A—C11A	−0.1 (14)
C1—B1—C11—C12	−0.9 (9)	C11B—B1B—C1B—C6B	177.9 (9)
Br1—B1—C11—C12	178.8 (7)	Br1B—B1B—C1B—C6B	−2.7 (14)
C16—C11—C12—C13	−2.3 (13)	C11B—B1B—C1B—C2B	−1.7 (9)
B1—C11—C12—C13	177.6 (7)	Br1B—B1B—C1B—C2B	177.6 (6)
C16—C11—C12—C2	−179.2 (7)	C6B—C1B—C2B—C3B	1.8 (11)
B1—C11—C12—C2	0.8 (9)	B1B—C1B—C2B—C3B	−178.5 (8)
C3—C2—C12—C13	4.1 (14)	C6B—C1B—C2B—C12B	−178.6 (7)
C1—C2—C12—C13	−176.8 (8)	B1B—C1B—C2B—C12B	1.1 (8)
C3—C2—C12—C11	−179.4 (9)	C1B—C2B—C3B—C4B	−2.3 (12)
C1—C2—C12—C11	−0.3 (10)	C12B—C2B—C3B—C4B	178.1 (8)
C11—C12—C13—C14	1.6 (12)	C2B—C3B—C4B—C5B	1.5 (12)
C2—C12—C13—C14	177.8 (8)	C3B—C4B—C5B—C6B	−0.1 (13)
C12—C13—C14—C15	−0.3 (13)	C4B—C5B—C6B—C1B	−0.5 (12)
C13—C14—C15—C16	−0.2 (14)	C2B—C1B—C6B—C5B	−0.3 (11)
C14—C15—C16—C11	−0.5 (13)	B1B—C1B—C6B—C5B	−180.0 (9)
C12—C11—C16—C15	1.8 (13)	C1B—B1B—C11B—C12B	1.7 (9)
B1—C11—C16—C15	−178.2 (8)	Br1B—B1B—C11B—C12B	−177.6 (6)
C11A—B1A—C1A—C6A	178.2 (9)	C1B—B1B—C11B—C16B	−179.9 (9)
Br1A—B1A—C1A—C6A	−1.7 (14)	Br1B—B1B—C11B—C16B	0.8 (14)
C11A—B1A—C1A—C2A	−2.0 (9)	C16B—C11B—C12B—C13B	0.9 (12)
Br1A—B1A—C1A—C2A	178.1 (6)	B1B—C11B—C12B—C13B	179.6 (7)
C6A—C1A—C2A—C3A	1.7 (12)	C16B—C11B—C12B—C2B	−179.7 (7)
B1A—C1A—C2A—C3A	−178.2 (7)	B1B—C11B—C12B—C2B	−1.0 (9)
C6A—C1A—C2A—C12A	−178.9 (7)	C3B—C2B—C12B—C13B	−1.2 (14)
B1A—C1A—C2A—C12A	1.2 (9)	C1B—C2B—C12B—C13B	179.2 (8)
C1A—C2A—C3A—C4A	−1.9 (12)	C3B—C2B—C12B—C11B	179.6 (8)
C12A—C2A—C3A—C4A	178.9 (8)	C1B—C2B—C12B—C11B	−0.1 (9)
C2A—C3A—C4A—C5A	0.3 (13)	C11B—C12B—C13B—C14B	0.9 (12)
C3A—C4A—C5A—C6A	1.6 (13)	C2B—C12B—C13B—C14B	−178.3 (8)
C4A—C5A—C6A—C1A	−1.8 (13)	C12B—C13B—C14B—C15B	−1.8 (12)
C2A—C1A—C6A—C5A	0.2 (12)	C13B—C14B—C15B—C16B	0.8 (12)
B1A—C1A—C6A—C5A	180.0 (9)	C14B—C15B—C16B—C11B	1.1 (12)
C1A—B1A—C11A—C16A	179.9 (9)	C12B—C11B—C16B—C15B	−1.9 (12)
Br1A—B1A—C11A—C16A	−0.2 (15)	B1B—C11B—C16B—C15B	179.8 (9)

## References

[bb1] Blessing, R. H. (1995). *Acta Cryst.* A**51**, 33–38. 10.1107/s0108767394005726 7702794

[bb2] Flack, H. D. (1983). *Acta Cryst.* A**39**, 876–881.

[bb3] Gross, U. & Kaufmann, D. (1987). *Chem. Ber.* **120**, 991–994.

[bb4] Kaufmann, L., Vitze, H., Bolte, M., Lerner, H.-W. & Wagner, M. (2008). *Organometallics*, **27**, 6215–6221.

[bb5] Mewes, J., Lerner, H.-W. & Bolte, M. (2009). *Acta Cryst.* E**65**, o451. 10.1107/S1600536809003614 PMC2968660 21582124

[bb6] Sheldrick, G. M. (2008). *Acta Cryst.* A**64**, 112–122. 10.1107/S0108767307043930 18156677

[bb7] Spek, A. L. (2009). *Acta Cryst.* D**65**, 148–155. 10.1107/S090744490804362X PMC2631630 19171970

[bb8] Stoe & Cie (2001). *X-AREA* and *X-RED* Stoe & Cie, Darmstadt, Germany.

